# Post-COVID-19 syndrome and diabetes mellitus: a propensity-matched analysis of the International HOPE-II COVID-19 Registry

**DOI:** 10.3389/fendo.2023.1167087

**Published:** 2023-05-16

**Authors:** Mohammad Abumayyaleh, Iván J. Núñez Gil, María C. Viana-LLamas, Sergio Raposeiras Roubin, Rodolfo Romero, Emilio Alfonso-Rodríguez, Aitor Uribarri, Gisela Feltes, Víctor Manuel Becerra-Muñoz, Francesco Santoro, Martino Pepe, Alex Fernando Castro Mejía, Jaime Signes-Costa, Adelina Gonzalez, Francisco Marín, Javier López-País, Edoardo Manzone, Olalla Vazquez Cancela, Carolina Espejo Paeres, Alvaro López Masjuan, Lazar Velicki, Christel Weiß, David Chipayo, Antonio Fernandez-Ortiz, Ibrahim El-Battrawy, Ibrahim Akin, HOPE COVID-19 investigators

**Affiliations:** ^1^ Department of Cardiology, Angiology, Haemostaseology and Medical Intensive Care, University Medical Center Mannheim, Medical Faculty Mannheim, Heidelberg University, Mannheim, Germany; ^2^ European Center for AngioScience (ECAS) and German Center for Cardiovascular Research (DZHK) partner site Heidelberg/Mannheim, Mannheim, Germany; ^3^ Hospital Clínico San Carlos, Universidad Complutense de Madrid, Instituto de Investigación, Sanitaria del Hospital Clínico San Carlos (IdISSC), Madrid, Spain; ^4^ Hospital Universitario Guadalajara, Guadalajara, Spain; ^5^ University Hospital Álvaro Cunqueiro, Vigo, Spain; ^6^ Hospital Universitario Getafe, Getafe, Universidad Europea, Madrid, Spain; ^7^ Hospital University of Bellvitge, Barcelona, Spain; ^8^ Cardiology Department, Vall d’Hebron University Hospital and Research Institute, Universitat Autonoma de Barcelona, Barcelona, Spain; ^9^ Centro de Investigacion Biomedica en Red para Enfermedades Cardiovasculares (CIBERCV), Madrid, Spain; ^10^ Hospital Nuestra Señora de América, Madrid, Spain; ^11^ Hospital Clinico Universitario Virgen de la Victoria, Malaga, Spain; ^12^ Department of Medical and Surgical Sciences, University of Foggia, Foggia, Italy; ^13^ Azienda Ospedaliero-Universitaria Consorziale Policlinico di Bari, Bari, Italy; ^14^ Hospital General del norte de Guayaquil IESS Los Ceibos, Guayaquil, Ecuador; ^15^ Hospital Clínico de Valencia, INCLIVA, Valencia, Spain; ^16^ Hospital Universitario Infanta Sofia, Madrid, Spain; ^17^ Hospital Clínico Universitario Virgen de la Arrixaca, Murcia, Spain; ^18^ Complejo Hospitalario Universitario de Ourense, Ourense, Spain; ^19^ Hospital del Sureste, Madrid, Spain; ^20^ Complejo Hospitalario Universitario de Santiago de Compostela, Santiago, Spain; ^21^ Hospital Universitario Príncipe de Asturias, Madrid, Spain; ^22^ Hospital Universitario Juan Ramón Jimenez, Huelva, Spain; ^23^ Faculty of Medicine, University of Novi Sad, Novi Sad, Serbia; ^24^ Institute of Cardiovascular Diseases Vojvodina, Sremska Kamenica, Serbia; ^25^ Department for Statistical Analysis, University Heidelberg, Mannheim, Germany; ^26^ Department of Cardiology and Angiology, Bergmannsheil University Hospitals, Ruhr University of Bochum, Bochum, Germany

**Keywords:** diabetes mellitus, post-COVID-19 syndrome, SARS-CoV-2, respiratory complications, reinfection, vaccination rate, long-term mortality

## Abstract

**Background:**

Diabetes mellitus (DM) is one of the most frequent comorbidities in patients suffering from severe acute respiratory syndrome coronavirus 2 (SARS-CoV-2) with a higher rate of severe course of coronavirus disease (COVID-19). However, data about post-COVID-19 syndrome (PCS) in patients with DM are limited.

**Methods:**

This multicenter, propensity score-matched study compared long-term follow-up data about cardiovascular, neuropsychiatric, respiratory, gastrointestinal, and other symptoms in 8,719 patients with DM to those without DM. The 1:1 propensity score matching (PSM) according to age and sex resulted in 1,548 matched pairs.

**Results:**

Diabetics and nondiabetics had a mean age of 72.6 ± 12.7 years old. At follow-up, cardiovascular symptoms such as dyspnea and increased resting heart rate occurred less in patients with DM (13.2% vs. 16.4%; *p* = 0.01) than those without DM (2.8% vs. 5.6%; *p* = 0.05), respectively. The incidence of newly diagnosed arterial hypertension was slightly lower in DM patients as compared to non-DM patients (0.5% vs. 1.6%; *p* = 0.18). Abnormal spirometry was observed more in patients with DM than those without DM (18.8% vs. 13; *p* = 0.24). Paranoia was diagnosed more frequently in patients with DM than in non-DM patients at follow-up time (4% vs. 1.2%; *p* = 0.009). The incidence of newly diagnosed renal insufficiency was higher in patients suffering from DM as compared to patients without DM (4.8% vs. 2.6%; *p* = 0.09). The rate of readmission was comparable in patients with and without DM (19.7% vs. 18.3%; *p* = 0.61). The reinfection rate with COVID-19 was comparable in both groups (2.9% in diabetics vs. 2.3% in nondiabetics; *p* = 0.55). Long-term mortality was higher in DM patients than in non-DM patients (33.9% vs. 29.1%; *p* = 0.005).

**Conclusions:**

The mortality rate was higher in patients with DM type II as compared to those without DM. Readmission and reinfection rates with COVID-19 were comparable in both groups. The incidence of cardiovascular symptoms was higher in patients without DM.

## Introduction

Coronavirus disease 2019 (COVID-19) is caused by severe acute respiratory syndrome coronavirus 2 (SARS-CoV-2) and is associated with significant morbidity and mortality (1).

Among other related diseases such as arterial hypertension and obesity, diabetes mellitus (DM) is identified as a risk factor for the severe course of COVID-19, developing sepsis, and mortality (2–4).

In patients suffering from COVID-19, SARS-CoV-2 binds the angiotensin-converting enzyme 2 (ACE2) receptor and uses it as a potential target for viral interventions (5). In diabetic mice, the expression of ACE2 is increased as compared to mice without DM. In addition, patients who suffered from insufficient glycemic control showed worse outcomes, such as more complications and higher mortality rates (6). New-onset DM and metabolic complications in patients suffering from manifested DM with high doses of insulin have been revealed in COVID-19 (7, 8). Furthermore, uncontrolled glycemic levels in DM patients cause organ injury and may be exacerbated in patients suffering from COVID-19 (9).

The international Health Outcome Predictive Evaluation for COVID-19 (HOPE COVID-19) Registry was initiated to investigate comorbidity and mortality of COVID-19 (10). In the Health Outcome Predictive Evaluation for COVID-19 II (HOPE-II COVID-19) Registry, we investigated readmission, reinfection, vaccination rate, cardiovascular, neuropsychiatric, respiratory, gastrointestinal, and other symptoms in hospitalized patients suffering from COVID-19 and concomitant DM type II. Complications related to COVID-19 and long-term mortality were also systematically analyzed.

## Material and methods

### Study design and participants

HOPE-II COVID-19 (NCT04334291) is an international project at 55 international centers. It is designed as a retrospective and prospective cohort registry to investigate post-COVID-19 syndrome without any conflict of interest. We included hospitalized patients with a confirmed diagnosis of COVID-19. There are no exclusion criteria, except for the patient’s explicit refusal to participate. Initially, data on 8,828 hospitalized patients suffering from COVID-19 were collected until 30th September 2021. In this study, we excluded 56 patients due to age <18 and 53 patients with DM type I. Data from 8,719 consecutive patients with COVID-19 regarding their concomitant DM type II status were analyzed.

### Ethics approval

This study was executed in compliance with the Declaration of Helsinki regarding human subjects, and the study was approved by the center ethics committee of Hospital Clinico San Carlos (Internal Code: 21/128-E) and, when needed, in all involved centers.

### DM type II

DM type II was known and diagnosed by medical physicians. Data were collected from the patient’s medical records.

### Post-COVID-19 syndrome

Patients suffering from post-COVID-19 syndrome describe new-onset symptoms following initial recovery from an acutely confirmed COVID-19 or ongoing from the initial illness. This condition occurs 3 months from the onset of COVID-19 with symptoms that last for at least 2 months and cannot be explained by an alternative diagnosis. Symptoms may also fluctuate or relapse over time (11).

### Outcomes and follow-up

We described long-term mortality as a primary endpoint. Readmission, reinfection rate, respiratory complications, cardiovascular, neuropsychiatric, respiratory, gastrointestinal, and other symptoms as secondary endpoints were also evaluated. Follow-up for the overall population for mortality was 20 months (mean post-COVID-19; 2.6 ± 4.6).

### Statistical analysis

Descriptive and comparative analyses were presented. Continuous variables were shown as mean ± standard deviation if the distribution was normal or median (min–max) if not. Categorical variables were presented as frequency rates and percentages. The Chi-square test was used for categorical variables for group comparisons. Quantitative variables were performed using the Mann–Whitney *U* test for nonparametric variables and the Student’s *t*-test for parametric variables, as verified by the Kolmogorov–Smirnov test. We applied a propensity score (PS)-based matching method to control for confounding baseline variables due to the nonrandomized nature of the study and the different participating centers. In a multivariable logistic regression test, hazard ratio (HR) with 95% confidence intervals (95% CI) was calculated for the determination of risk factors for the endpoint. Predictors of mortality were identified by univariate analysis. Predictors with *p* < 0.05 were analyzed by logistic multivariable regression. The multivariable regression test was used to investigate predictors of mortality, adjusting for all significant variables: age; male as sex; obesity; comorbidities such as arterial hypertension, dyslipidemia, DM type II, renal insufficiency, heart disease, cerebrovascular disease, liver disease, and cancer disease; immunosuppression; home oxygen therapy; premedication; symptomatic; clinical parameters such as peripheral oxygen saturation (SpO_2_) <92% and reduced blood pressure (systolic blood pressure <90 mmHg or diastolic blood pressure <60 mmHg); and laboratory parameters. *p*-value of <0.05 was recognized as statistically significant. Statistical analysis was performed with IBM SPSS Statistics version 27.

## Results

### Baseline characteristics and in-hospital complications

Data from 8,719 consecutive hospitalized patients (*n* = 1,578 with DM; *n* = 7,141 with non-DM) with confirmed COVID-19 were collected. The 1:1 propensity score matching (PSM) according to age and sex resulted in 1,548 matched pairs. The mean age of matched pairs was 72.6 ± 12.7 years old. Even more, the male sex was 63.5% in both groups. Diabetics suffered from more chronic conditions such as arterial hypertension (77.5% vs. 58.5%; *p* < 0.0001), renal insufficiency (13.6% vs. 8.1%; *p* < 0.0001), and liver disease (5.7% vs. 3.4%; *p* = 0.002). In-hospital complications were observed more in diabetics as compared to nondiabetics, for example, respiratory insufficiency (62.1% vs. 56.3%; *p* = 0.001), acute kidney injury (26.6% vs. 19.8%; *p* < 0.0001), and sepsis (15.4% vs. 12.8%, *p* = 0.04). Other baseline characteristics, immunosuppression, home oxygen therapy, premedication, symptomatic, clinical, and laboratory parameters, in-hospital complications, and intervention procedures during hospitalization are presented in **Table 1**.

**Table 1:** Patients with diabetes mellitus type II as compared to patients without DM II, baseline characteristics, laboratory and radiographic findings, complications, and clinical outcomes.

Characteristic	Diabetics (*N* = 1,548)	Nondiabetics (*N* = 1,548)	*p*-value^*^
Age (mean ± SD (years))	72.6 ± 12.7	72.6 ± 12.7	1.00
Male as sex (no. (%))	983 (63.5)	983 (63.5)	1.00
Chronic conditions (no. (%))			
**Arterial hypertension**	1,200 (77.5)	906 (58.5)	**<0.0001**
**Dyslipidemia**	927 (59.9)	551 (35.6)	**<0.0001**
**Obesity**	486 (31.4)	221 (14.3)	**<0.0001**
**Current smoking**	86 (6.2)	83 (5.9)	0.73
Renal insufficiency^a^	211 (13.6)	126 (8.1)	**<0.0001**
**Lung disease**	362 (30.1)	329 (28)	0.26
**Cardiac disease**	538 (34.8)	414 (26.7)	**<0.0001**
**Cerebrovascular disease**	192 (12.4)	164 (10.6)	0.12
**Connective tissue disease**	48 (3.1)	43 (2.8)	0.60
**Liver disease**	88 (5.7)	52 (3.4)	**0.002**
**Cancer disease**	291 (18.8)	237 (15.3)	**0.009**
Immunosuppression^b^	134 (8.7)	112 (7.2)	0.14
Home oxygen therapy	74 (4.8)	71 (4.6)	0.80
Premedication (no. (%))			
**ASA**	453 (29.3)	263 (17)	**<0.0001**
**Antiplatelet drug**	119 (7.7)	75 (4.8)	**0.001**
**Oral anticoagulation**	251 (16.2)	220 (14.2)	0.12
**Beta-blockers**	420 (27.1)	287 (18.5)	**<0.0001**
**ACEI/ARB**	863 (55.8)	638 (41.2)	**<0.0001**
Symptomatic (no. (%))			
**Asymptomatic**	81 (5.2)	105 (6.8)	0.07
**Dyspnea**	961 (62.9)	911 (59.5)	**0.05**
**Tachypnea > 22 breaths/min**	485 (31.4)	455 (29.4)	0.24
**Hemoptysis**	26 (1.7)	32 (2.1)	0.42
**Fatigue**	727 (47)	718 (46.4)	0.75
**Anosmia/hyposmia**	55 (3.6)	67 (4.3)	0.27
**Dysgeusia**	66 (4.3)	73 (4.7)	0.54
**Sore throat**	117 (7.6)	159 (10.3)	**0.01**
**Fever**	1,102 (71.3)	1,150 (74.4)	0.06
**Cough**	950 (61.5)	944 (61.1)	0.83
**Vomiting**	107 (6.9)	95 (6.1)	0.38
**Diarrhea**	268 (17.3)	234 (15.1)	0.10
**Erythromelalgia**	369 (23.9)	443 (28.7)	**0.003**
Clinical parameters (no. (%))			
**Peripheral oxygen saturation < 92%**	690 (44.6)	604 (39.1)	**0.002**
Abnormal blood pressure^c^	139 (9.9)	116 (8.3)	0.13
GCS < 15	149 (11.8)	144 (11)	0.62
Laboratory parameters (no. (%) or median (min–max))			
**Elevated D-dimer**	953 (61.6)	903 (58.4)	0.07
**Elevated procalcitonin**	302 (19.5)	231 (14.9)	**0.0007**
**Elevated CRP**	1,382 (89.4)	1,343 (86.9)	**0.03**
**Elevated TnI**	206 (13.3)	165 (10.7)	**0.02**
Elevated transaminases^d^	505 (32.7)	579 (37.5)	**0.006**
**Elevated ferritin**	494 (32)	515 (33.3)	0.42
**Elevated triglyceride**	172 (11.1)	129 (8.3)	**0.009**
**Elevated LDH**	1,018 (65.9)	1,033 (66.8)	0.59
**Creatinine (mg/dl)**	1.02 (0.38–11.3)	0.96 (0.12–33.9)	**0.0005**
**Leukocytes (10E9/L)**	7,000 (550–90,004)	6,440 (440–88,400)	**<0.0001**
**Lymphocytes (10E9/L)**	960 (12–41,100)	930 (244–77,100)	0.25
**Hemoglobin (g/dl)**	13 (1–19.3)	14 (4–18)	**<0.0001**
**Thrombocytes (10E9/L)**	201,000 (13,000–716,000)	190,000 (10,000–980,000)	**<0.0001**
**Natrium level (mmol/L)**	137 (115–179)	138 (117–180)	**<0.0001**
In-hospital complication			
**Respiratory insufficiency**	958 (62.1)	867 (56.3)	**0.001**
**Heart failure**	191 (12.4)	128 (8.3)	**0.0002**
**Acute kidney injury**	411 (26.6)	305 (19.8)	**<0.0001**
**Upper respiratory tract infection**	257 (16.7)	240 (15.6)	0.41
**Pneumonia**	1,344 (89.4)	1,336 (88.4)	0.41
**SIRS**	389 (25.2)	355 (23)	0.16
**Sepsis**	238 (15.4)	197 (12.8)	**0.04**
Any relevant bleeding^e^	65 (4.2)	46 (3)	0.07
**Embolic event**	49 (3.2)	47 (3.1)	0.84
Oxygen therapy			
**O_2_ at the admission**	1,238 (80.2)	1,157 (75.1)	**0.0007**
**High-flow nasal cannula**	347 (22.5)	336 (21.8)	0.65
**Noninvasive mechanical ventilation**	250 (16.2)	237 (15.4)	0.54
**Invasive mechanical ventilation**	163 (10.6)	123 (8)	**0.01**
Another medication or intervention procedures during the admission			
**Prone position**	196 (12.7)	169 (11)	0.14
**ECMO**	119 (7.7)	82 (5.3)	**0.007**
**Use of glucocorticoids**	546 (35.4)	526 (34.1)	0.47
**Use of hydroxychloroquine**	1,173 (76)	1,180 (76.6)	0.69
Use of antiviral drugs^f^	714 (46.2)	812 (53)	**0.0003**
**Use of interferon**	180 (11.7)	233 (15.1)	**0.005**
**Use of tocilizumab**	131 (8.5)	128 (8.3)	0.85
**Use of antibiotics**	1,181 (76.5)	1,113 (72.2)	**0.007**
ACEI/ARB^g^	476 (30.9)	354 (23)	**<0.0001**
**Anticoagulation**	856 (81.7)	791 (75.8)	**0.001**
Discharge			
**ACEI/ARB**	82 (30.8)	71 (24.7)	0.11
**Antiplatelet drug**	226 (14.7)	147 (9.6)	**<0.0001**
**Anticoagulation**	413 (26.8)	365 (23.7)	**0.05**

ASA, acetylsalicylic acid; ACEI/ARB, angiotensin-converting enzyme inhibitor/angiotensin-receptor blocker; CRP, C-reactive protein; GCS, Glasgow coma scale; ECMO, extracorporeal membrane oxygenation; SIRS, systemic inflammatory response syndrome; TnI, high-sensitivity troponin I (cardiac injury; troponin > 99th percentile upper reference limit).

a CrCL < 30.

b Immunosuppressive therapy for psoriatic arthritis, lung transplantation, kidney transplantation, or systemic lupus erythematosus; oncological diseases such as mamma-ca, prostate-ca, myelodysplastic syndrome, or gammopathy; glucocorticoid therapy caused by COPD; dialysis; HIV; or hepatitis.

c Systolic blood pressure < 90 mmHg or diastolic blood pressure < 60 mmHg.

d ALAT and ASAT.

e Rectorrhagia, hematuria, epistaxis, and popliteal aneurysm bleeding with relevant decreased hemoglobin > 2 mg/l.

f Lopinavir or/and ritonavir.

g Premedication with ACEI/ARB is not stopped.*Statistical significance level is set at 0.05 and value of statistical significance is emphasized in bold.

### Clinical outcomes at long-term follow-up

Mean follow-up (2.6 ± 4.6 months) data were available for 412 diabetics and 443 nondiabetics. The readmission rate due to any cause was similar in diabetics and nondiabetics, respectively (19.7% vs. 18.3%; *p* = 0.61). The reinfection rate with COVID-19 was also comparable in patients with DM than those without DM (2.9% vs. 2.3%; *p* = 0.55). Additionally, diabetics were vaccinated more than nondiabetics at follow-up with the same time to vaccination (11.9 ± 3.1 months in diabetics vs. 12.2 ± 2.9 months in nondiabetics) (57.3% vs. 51.7%; *p* = 0.10). At follow-up, cardiovascular symptoms such as dyspnea and an increase in resting heart rate after discharge occurred less frequently in patients suffering from DM (13.2% vs. 16.4%; *p* = 0.01) than those without DM (2.8% vs. 5.6%; *p* = 0.05), respectively. In addition, the mortality rate at the 20-month follow-up was significantly higher in DM than in non-DM patients (33.9% vs. 29.1%; *p* = 0.005). Cardiovascular, neuropsychiatric, respiratory, gastrointestinal, and other symptoms are presented in **Table 2**.

**Table 2:** Follow-up in patients suffering from DM type II as compared to those without DM.

	Diabetics (*N* = 1,548)	Nondiabetics (*N* = 1,548)	*p*-value^*^
Follow-up (mean ± SD)			
**Follow-up time (months (PCS))**	2.6 ± 4.6	2.8 ± 4.9	0.77
**Duration to recovery (months)**	2.2 ± 4.6	2.4 ± 4.9	0.51
**Duration to readmission (months)**	2.5 ± 4.5	2.6 ± 4.6	0.95
**Number of patients (*n*)**	412	443	–
**Readmission**	81 (19.7)	81 (18.3)	0.61
**Vaccination**	236 (57.3)	229 (51.7)	0.10
**Time to vaccination (months)**	11.9 ± 3.1	12.2 ± 2.9	0.74
**Reinfection with COVID-19**	12 (2.9)	10 (2.3)	0.55
**Clinical event after discharge**	171 (43.1)	181 (42)	0.75
Cardiovascular symptoms			
**Fatigue**	114 (28.7)	125 (29)	0.93
**Dyspnea**	204 (13.2)	254 (16.4)	**0.01**
**Dizziness**	34 (8.6)	35 (8.1)	0.82
**Chest pain**	28 (7.1)	28 (6.5)	0.75
**Acute coronary syndrome**	3 (0.8)	4 (0.9)	1.00
**Palpitation**	24 (6.1)	37 (8.6)	0.16
**Increase in resting heart rate**	11 (2.8)	24 (5.6)	**0.05**
**Syncope**	2 (0.5)	8 (1.9)	0.11
**Arrhythmias**	27 (6.8)	22 (5.1)	0.30
**Atrial fibrillation**	21 (5.3)	26 (6)	0.65
**Perimyocarditis**	1 (0.3)	2 (0.5)	1.00
**Limb edema**	13 (3.3)	18 (4.2)	0.50
**New hypertension**	2 (0.5)	7 (1.6)	0.18
**New left ventricular dysfunction**	5 (1.3)	7 (1.6)	0.66
**Relevant bleeding**	5 (1.3)	5 (1.2)	0.90
Neuropsychiatric symptoms			
**Headache**	11 (2.8)	21 (4.9)	0.12
**Migraine**	5 (1.3)	11 (2.6)	0.18
**Ageusia**	17 (4.3)	19 (4.4)	0.93
**Anosmia**	12 (3)	18 (4.2)	0.38
**Attention disorder**	16 (4)	25 (5.8)	0.24
**Memory loss**	31 (7.8)	34 (7.9)	0.97
**Cognitive disorder**	18 (4.5)	20 (4.6)	0.94
**Anxiety**	34 (8.6)	54 (12.5)	0.06
**Depression**	26 (6.6)	35 (8.1)	0.39
**Tinnitus or hearing loss**	9 (2.3)	14 (3.3)	0.39
**Sleeping disorder**	27 (6.8)	36 (8.4)	0.40
**Mood disorder**	22 (5.5)	31 (7.2)	0.33
**Paranoia**	16 (4)	5 (1.2)	**0.009**
Respiratory symptoms			
**Cough**	33 (8.3)	42 (9.7)	0.47
**Reduce pulmonary diffusing capacity**	28 (7.1)	44 (10.2)	0.11
**Polypnea**	15 (3.8)	19 (4.4)	0.65
**Sleep apnea**	13 (3.3)	9 (2.1)	0.29
Gastrointestinal symptoms			
**Tongue involvement**	1 (0.3)	7 (1.6)	0.07
**Digestive disorder**	20 (5)	17 (3.9)	0.45
**Nausea/vomiting**	10 (2.5)	8 (1.9)	0.51
Other symptoms			
**Intermittent fever**	8 (2)	10 (2.3)	0.76
**Chills**	6 (1.5)	8 (1.9)	0.70
**Hair loss**	20 (5)	18 (4.2)	0.55
**Joint pain**	19 (4.8)	25 (5.8)	0.52
**Myalgia**	26 (6.6)	32 (7.4)	0.62
**Sweat**	5 (1.3)	4 (0.9)	0.74
**Weight loss**	24 (6.1)	23 (5.3)	0.66
**Cutaneous involvement**	6 (1.5)	13 (3)	0.15
**New diabetes**	–	4 (0.9)	–
**New renal insufficiency**	19 (4.8)	11 (2.6)	0.09
**Pain**	12 (3)	8 (1.9)	0.28
**Red eyes**	4 (1)	6 (1.4)	0.76
**Flushing**	4 (1)	2 (0.5)	0.43
**Incident neoplasia**	2 (0.5)	6 (1.4)	0.29
Management after discharge			
**Home oxygen therapy**	43 (10.8)	37 (8.6)	0.27
**ASA**	99 (24.9)	58 (13.5)	**<0.0001**
**Antiplatelet drug**	34 (8.6)	23 (5.3)	0.07
**Anticoagulation**	69 (17.4)	54 (12.5)	**0.05**
**ACEI/ARB**	140 (35.3)	113 (26.2)	**0.005**
**Beta-blockers**	75 (18.9)	69 (16)	0.27
**Beta agonist inhalation therapy**	34 (8.6)	46 (10.7)	0.31
**Vitamin supplementation**	72 (18.1)	80 (18.6)	0.88
**Antidepressant**	47 (11.8)	64 (14.9)	0.20
**Statin**	151 (38)	103 (23.9)	**<0.0001**
Diagnostic test after discharge			
**Elevated di-dimer**	137 (34.6)	151 (35.2)	0.86
**Elevated CRP**	167 (42.2)	183 (42.7)	0.89
**Elevated procalcitonin**	45 (11.4)	34 (7.9)	0.09
**Elevated TnI**	18 (4.6)	16 (3.7)	0.56
**Elevated NT-proBNP**	23 (5.8)	29 (6.8)	0.57
**Elevated transaminases^a^**	92 (23.2)	100 (23.3)	0.98
**Abnormal spirometry**	21 (18.8)	17 (13)	0.24
**Any chest X-ray abnormality**	99 (39.4)	103 (38.9)	0.99
**Any CT abnormality**	37 (35.6)	48 (35.3)	0.60
**In-hospital mortality**	492 (31.8)	426 (27.5)	**0.009**
**Long-term mortality**	524 (33.9)	451 (29.1)	**0.005**

PCS, post-COVID-19 syndrome; ASA, acetylsalicylic acid; ACEI/ARB, angiotensin-converting enzyme inhibitor/angiotensin-receptor blocker; CRP, C-reactive protein; TnI, high-sensitivity troponin I cardiac injury; troponin > 99th percentile upper reference limit. ^a^ALAT and ASAT.*Statistical significance level is set at 0.05 and value of statistical significance is emphasized in bold.Summarized, - means not available.

### PSM and predictors of mortality

**Figure 1:**
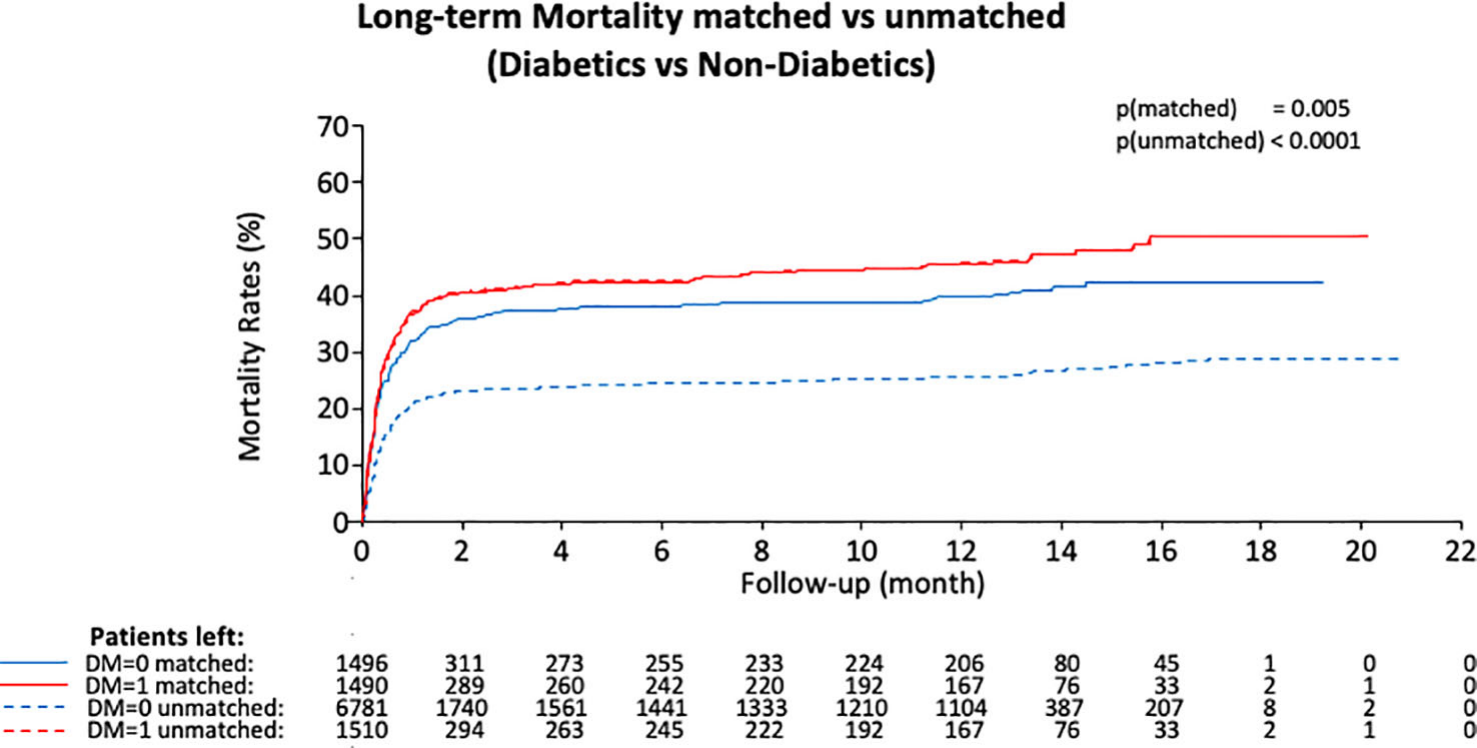
Kaplan–Meier curve for long-term mortality divided by diabetics vs. nondiabetics in the overall population and matched cohort. In both comparisons, a worse outcome in diabetics was detected.

The mortality rate at long-term follow-up was significantly higher in patients with DM than those without, in the overall cohort and in the matched cohort, respectively (*p* < 0.0001 and *p* = 0.005). The Kaplan–Meier curve with landmark analysis is displayed in **Figure 1**. In the multivariable analysis for mortality, age, and male sex were determined as predictors for mortality, respectively (HR: 2.34; *p* < 0.0001) (HRK 1.23; *p* = 0.008). Other predictors are performed in **Table 3**. Clinical outcomes before PSM are presented in the **Supplementary Appendix**.

**Table 3:** Predictors of mortality, multivariable analysis.

Variable	Univariable analysis		Multivariable analysis	
	HR	*p*-value	HR	*p*-value
Patient demographics				
**Age ≥70**	2.90	**<0.0001**	2.34	**<0.0001**
**Male**	1.19	**0.01**	1.23	**0.008**
Chronic conditions				
**Dyslipidemia**	1.12	0.07		
**Diabetes mellitus**	1.18	**0.01**		
**Obesity**	1.01	0.88		
**Renal insufficiency**	1.86	**<0.0001**	1.33	**0.003**
**Cancer disease**	1.46	**<0.0001**		
Immunosuppression	1.41	**0.0009**	1.40	**0.003**
Premedication				
**ASA**	1.38	**<0.0001**		
**Oral anticoagulation**	1.66	**<0.0001**		
Clinical parameters				
**SpO_2_ < 92%** ^a^	3.14	**<0.0001**	2.13	**<0.0001**
Abnormal blood pressure^b^	2.09	**<0.0001**	1.36	**0.002**
GCS < 15	2.67	**<0.0001**	1.50	**<0.0001**
Clinical presentation				
**Dyspnea**	1.48	**<0.0001**		
**Tachypnea > 22 breaths/min**	2.17	**<0.0001**	1.41	**<0.0001**
**Dysgeusia**	0.32	**<0.0001**	0.40	**0.001**
**Sore throat**	0.79	0.07		
**Cough**	0.77	**<0.0001**	0.84	**0.02**
**Erythromelalgia**	0.73	**<0.0001**		
Laboratory parameters				
**Elevated procalcitonin**	1.89	**<0.0001**	1.53	**<0.0001**
**Elevated CRP**	1.60	**<0.0001**		
**Elevated LDH**	1.50	**<0.0001**	1.20	**0.04**

HR, hazard ratio; ASA, acetylsalicylic acid; SpO_2_, peripheral oxygen saturation; GCS, Glasgow coma scale.

a SpO_2_ < 92% at admission.

b Systolic blood pressure < 90 mmHg or diastolic blood pressure < 60 mmHg.Statistical significance level is set at 0.05 and value of statistical significance is emphasized in bold.

## Discussion

This study presents characteristics of PCS in patients suffering from DM as compared to those without DM. The main findings of this study are as follows: (1) readmission rate for any cause was similar in diabetics than nondiabetics at follow-up; (2) reinfection rate with COVID-19 was similar in both groups; (3) symptoms such as dyspnea and an increase of resting heart rate occurred less in diabetics as compared to nondiabetics; (4) The incidence of newly diagnosed arterial hypertension was less in diabetics than nondiabetics without statistical significance; (5) respiratory complications were revealed in diabetics and nondiabetics; and (5) long-term mortality was higher in patients suffering from DM as compared to those without DM.

Recently, it has been reported that the progression of type II DM is associated with increased insulin resistance accompanied by chronic inflammation and endothelial and ß-cell dysfunction (12). On the other hand, the inflammatory response in infected patients with SARS-CoV-2 may worsen insulin resistance and endothelial dysfunction (13). The existence of both diseases may further enhance the inflammation and decrease interferon levels, neutrophil chemotaxis, and T lymphocyte-mediated immune response with impairment of cytokine production (14–16). That is associated with a severe course of COVID-19 in DM patients. Furthermore, ACE2 expression increases insulin resistance. This receptor and dipeptidyl peptidase 4 (DPP4), which may be a factor in the severity of COVID-19 infection, are present in several physiological processes and are modulated by hyperglycemia and pharmacological therapies that are common in DM patients (17). In addition, chronic hyperglycemia leads to chronic vascular and kidney disease. Other comorbidities, such as obesity and hypertension, are present in concurrent DM. These diabetes-related comorbidities may negatively impact outcomes in DM patients with COVID-19 (18, 19).

### DM as a risk factor for post-COVID-19 syndrome

Our DM cohort had more comorbidities such as arterial hypertension, renal insufficiency, liver disease, and cardiac disease than patients without DM. Furthermore, respiratory insufficiency requiring oxygen therapy and invasive mechanical ventilation (MV) was observed more in diabetics as compared to nondiabetics. During hospitalizations, sepsis and acute kidney injury occurred more often in diabetics than nondiabetics. A prospective study showed that the persistence of symptoms was associated with the severity of the disease at the beginning and that the intensive care unit (ICU) admission was an independent risk factor for PCS (20). In addition, the need for MV was determined as a predictor for the development of PCS (21). However, it has been reported that 60% of low-risk patients for mortality with COVID-19 suffered from severe PCS (22). In patients with DM, optimizing hyperglycemia therapy improve metabolic function which may be beneficial for the long-term management of patients with PCS (23). In this study, PCS was slightly comparable despite the different comorbidities and in-hospital complications in both groups.

### Cardiovascular symptoms

In our study, dyspnea and an increase in resting heart rate occurred more significantly in nondiabetics as compared to diabetics. Additionally, newly diagnosed arterial hypertension was also revealed slightly more in nondiabetics than diabetics. Regarding that, the persistence of cardiovascular symptoms was recently reported (24). In one of the studies from Wuhan, Huang et al. showed that patients infected with SARS-CoV-2 suffered from acute cardiac injury (25). Subclinical myocarditis with an increased risk of arrhythmias may play a role in PCS (26). Data about the comparison between diabetics and nondiabetics are limited.

### Neuropsychiatric symptoms

This study presented neuropsychiatric symptoms generally more common in nondiabetics as compared to diabetics without statistical significance, for example, headache, sleeping disorder, and anxiety. However, paranoia was observed significantly more in diabetics than nondiabetics at a 3-month follow-up. Studies reported that headache and other neuropsychiatric symptoms occurred after 3 months in patients infected with SARS-CoV-2 (27, 28). Guedj et al. reported that more areas in the brain showed hypometabolism in patients with PCS as compared to healthy subjects (29). Controlled, randomized studies are needed to investigate the neuropsychiatric symptoms in patients with DM as compared to those without DM.

### Respiratory symptoms

Renal insufficiency and cardiac disease were observed more in patients with DM than non-DM, while the rate of lung diseases was similar in matched pairs. At follow-up, our data presented a similar rate of sleep apnea in diabetics and nondiabetics. Furthermore, computer tomography (CT) and chest X-ray abnormalities were revealed in both groups, but dyspnea occurred significantly more in nondiabetics as compared to diabetics at follow-up. In one retrospective study with 77 days of follow-up, spirometry (9.3%) and chest radiology (19%) abnormalities were detected in 277 patients, of whom 51% had PCS (30). In 22 patients after COVID-19-associated acute respiratory distress syndrome (ARDS), signs of lung fibrosis were detected in 55% of patients at 3-month follow-up (31). In patients with critical COVID-19, 9.5% of patients needed home oxygen therapy after discharge at a 1-year follow-up (32). Furthermore, DM was identified as a risk factor for the requirement of oxygen therapy in patients suffering from COVID-19 (33). In our multivariable analysis, DM was not identified as a predictor for mortality.

This study has some limitations. It has a retrospective character; not all laboratory tests were done on all patients. Furthermore, data on hemoglobin A1c (HbA1c), antihyperglycemic treatment including metformin and DPP-4 inhibitors, and statin therapy at baseline are missing. A strength of our study is the sample size of patients with COVID-19 and concomitant DM type II at 55 international centers. The results are therefore real-world evidence.

To summarize, PCS was observed in diabetics and nondiabetics. However, the mortality rate was higher in diabetics as compared to nondiabetics. DM was not determined as a risk factor for mortality at follow-up.

## Data availability statement

The raw data supporting the conclusions of this article will be made available by the authors, without undue reservation.

## Ethics statement

This study was executed in compliance with the Declaration of Helsinki regarding in human subjects and the study was approved by the center Ethics Committee of Hospital Clinico San Carlos (Internal Code: 21/128-E) and, when needed, in all involved centers. Written informed consent for participation was not required for this study in accordance with the national legislation and the institutional requirements.

## Author contributions

MA, IG, IE-B, and IA made substantial contributions to the study’s concept and design. All authors obtained ethical approval. Data were collected by MA, IG, MV-L, SR, RR, EA-R, AU, GF, VB-M, FS, MP, AM, JS-C, AG, FM, JL-P, EM, OC, CP, AM, LV, DC, AF-O, MA, and CW analyzed all the data. CW supported the descriptive statistics. IJNG and IA approved the statistical analysis. MA, IG, IE-B, and IA prepared the manuscript. All authors contributed to the article and approved the submission version.
