# Supplementary material for: Post-COVID-19 syndrome and diabetes mellitus: a propensity-matched analysis of the International HOPE-II COVID-19 Registry
Source: Front Endocrinol (Lausanne). 2023 May 16;14:1167087. doi: 10.3389/fendo.2023.1167087 (PMC10227507; doi:10.3389/fendo.2023.1167087)
Supplement: Supplementary file 1 [file Table_1.pdf]

Supplementary Appendix

Table legends

Table S1: Follow-Up in Patients suffering from DM type II as compared to those without DM before propensity score matching

Table S1: Follow-Up in Patients suffering from DM type II as compared to those without DM before propensity score matching

	**Diabetics**<br>**N=1578**	**Non-diabetics**<br>**N=7141**	**P value***
**Follow-up; mean ± SD**			
Follow-up time in months (PCS)	2.2±4.6	3±5.2	
Duration to recovery in months	1.9±3.6	1.9±3.6	0.85
Duration to readmission in months	2.4±4.4	3±5	**<0.001**
Readmission	82/422 (19.4)	326/2371 (13.7)	**0.002**
Vaccination	238/422 (56.4)	1104/2371 (46.6)	**<0.001**
Time to vaccination in months	12±3	12±3	0.57
Reinfection with COVID-19	12/422 (2.8)	58/2372 (2.4)	0.63
Clinical event after discharge	173/407 (42.5)	944/2327 (40.6)	0.46
**Cardiovascular symptoms**			
Fatigue	114/407 (28)	621/2327 (26.7)	0.58
Dyspnea	205/422 (48.6)	1360/2372 (57.3)	**<0.001**
Dizziness	34/407 (8.4)	161/2328 (6.9)	0.29
Chest pain	28/407 (6.9)	173/2328 (7.4)	0.69
Acute coronary syndrome	3/407 (0.7)	27/2328 (1.2)	0.45
Palpitation	24/407 (5.9)	208/2328 (8.9)	**0.04**
Increase of resting heart rate	11/407 (2.7)	112/2328 (4.8)	0.06
Syncope	2/407 (0.5)	27/2328 (1.2)	0.17
Arrhythmias	27/407 (6.6)	130/2328 (5.6)	0.40
Atrial fibrillation	21/407 (5.2)	99/2328 (4.3)	0.41
Peri myocarditis	1/407 (0.2)	14/2328 (0.6)	0.32

Limb oedema	13/407 (3.2)	73/2328 (3.1)	0.95
New hypertension	2/407 (0.5)	59/2328 (2.5)	**0.003**
New left ventricular dysfunction	5/407 (1.2)	41/2328 (1.8)	0.44
Relevant bleeding	5/407 (1.2)	19/2327 (0.8)	0.41
**Neuropsychiatric symptoms**			
Headache	11/407 (2.7)	140/2328 (6)	**0.007**
Migraine	5/407 (1.2)	61/2328 (2.6)	0.09
Ageusia	17/407 (4.2)	109/2328 (4.7)	0.65
Anosmia	12/407 (2.9)	96/2328 (4.1)	0.26
Attention disorder	16/407 (3.9)	106/2328 (4.6)	0.58
Memory loss	31/407 (7.6)	130/2328 (5.6)	0.11
Cognitive disorder	18/407 (4.4)	79/2328 (3.4)	0.30
Anxiety	34/407 (8.4)	249/2328 (10.7)	0.15
Depression	26/407 (6.4)	164/2328 (7)	0.63
Tinnitus or hearing loss	9/407 (2.2)	51/2328 (2.2)	0.97
Sleeping disorder	27/407 (6.6)	177/2328 (7.6)	0.49
Mood disorder	22/407 (5.4)	128/2328 (5.5)	0.94
Paranoia	16/407 (3.9)	30/2327 (1.3)	**<0.001**
**Respiratory symptoms**			
Cough	33/407 (8.1)	239/2328 (10.3)	0.18
Reduce pulmonary diffusing capacity	28/407 (6.9)	167/2328 (7.2)	0.83
Polypnea	15/407 (3.7)	59/2328 (2.5)	0.19
Sleep apnea	13/407 (3.2)	30/2327 (1.3)	**0.004**
**Gastrointestinal symptoms**			

Tongue involvement	1/407 (0.2)	32/2328 (1.4)	**0.03**
Digestive disorder	20/407 (4.9)	107/2328 (4.6)	0.78
Nausea/Vomiting	10/407 (2.5)	47/2327 (2)	0.57
**Other symptoms**			
Intermittent fever	8/407 (2)	47/2328 (2)	0.94
Chills	6/407 (1.5)	41/2328 (1.8)	0.68
Hair loss	20/407 (4.9)	102/2328 (4.4)	0.63
Joint pain	19/407 (4.7)	143/2328 (6.1)	0.25
Myalgia	26/407 (6.4)	163/2328 (7)	0.65
Sweat	5/407 (1.2)	25/2328 (1.1)	0.78
Weight loss	24/407 (5.9)	108/2328 (4.6)	0.28
Cutaneous involvement	6/407 (1.5)	63/2328 (2.7)	0.14
New diabetes	-	29/2328 (1.2)	**-**
New renal insufficiency	19/407 (4.7)	52/2328 (2.2)	**0.004**
Pain	12/407 (2.9)	39/2328 (1.7)	0.08
Red eyes	4/407 (1)	27/2328 (1.2)	1.00
Flushing	4/407 (1)	6/2328 (0.3)	**0.05**
Incident neoplasia	2/407 (0.5)	21/2327 (0.9)	0.56
Management after discharge			
Home oxygen therapy	43/407 (10.6)	126/2327 (5.4)	**<0.001**
ASA	99/407 (24.3)	201/2327 (8.6)	**<0.001**
Antiplatelet Drug	34/407 (8.4)	77/2327 (3.3)	**<0.001**
Anticoagulation	69/407 (17)	241/2326 (10.4)	**<0.001**
ACEI/ARB's	140/407 (34.4)	442/2327 (19)	**<0.001**
Beta Blockers	75/407 (18.4)	284/2327 (12.2)	**0.001**

Beta Agonist Inhalation Therapy	34/407 (8.4)	188/2327 (8.1)	0.85
Vitamin supplementation	72/407 (17.7)	301/2327 (12.9)	**0.01**
Antidepressant	47/407 (11.5)	266/2327 (11.4)	0.95
Statin	151/407 (37.1)	360/2326 (15.5)	**<0.001**
Diagnostic test after discharge			
Elevated Di-Dimer	137/406 (33.7)	626/2320 (27)	**0.005**
Elevated CRP ∂	167/406 (41.1)	814/2320 (35.1)	**0.02**
Elevated Procalcitonin	45/406 (11.1)	167/2320 (7.2)	**0.007**
Elevated TnI ∞	18/406 (4.4)	65/2320 (2.8)	0.08
Elevated NT-proBNP	23/406 (5.7)	90/2320 (3.9)	0.09
Elevated Transaminases •	92/406 (22.7)	556/2320 (23.9)	0.79
Abnormal spirometry	21/112 (18.8)	76/905 (8.4)	**<0.001**
Any chest x-ray abnormality	99/251 (39.4)	453/1547 (29.3)	**0.001**
Any CT abnormality	37/104 (35.6)	197/857 (23)	**0.005**
In-hospital mortality	505/1578 (32)	1218/7138 (17.1)	**<0.001**
Long-term mortality	537/1578 (34)	1304/7138 (18.3)	**<0.001**

PCS post COVID-19 syndrome. Ω Acetylsalicylic acid. ∂ Angiotensin-converting enzyme inhibitor/ Angiotensin-recteptor blocker. ∂ C-reactive Protein. ∞ High sensitive Troponin I (cardiac injury; troponin > 99th percentile upper reference limit). • ALAT and ASAT.
